# Design and methods for a cluster randomized trial of the Sunless Study: A skin cancer prevention intervention promoting sunless tanning among beach visitors

**DOI:** 10.1186/1471-2458-9-50

**Published:** 2009-02-05

**Authors:** Sherry L Pagoto, Kristin L Schneider, Jessica Oleski, Jamie S Bodenlos, Philip Merriam, Yunsheng Ma

**Affiliations:** 1 Department of Medicine, Division of Preventive and Behavioral Medicine, University of Massachusetts Medical School, 55 Lake Avenue North, Worcester, MA, USA

## Abstract

**Background:**

Skin cancer is the most prevalent yet most preventable cancer in the US. While protecting oneself from ultraviolet radiation (UVR) can largely reduce risk, rates of unprotected sun exposure remain high. Because the desire to be tan often outweighs health concerns among sunbathers, very few interventions have been successful at reducing sunbathing behavior. Sunless tanning (self-tanners and spray tans), a method of achieving the suntanned look without UVR exposure, might be an effective supplement to prevention interventions.

**Methods and Design:**

This cluster randomized trial will examine whether a beach-based intervention that promotes sunless tanning as a substitute for sunbathing and includes sun damage imaging and sun safety recommendations is superior to a questionnaire only control group in reducing sunbathing frequency. Female beach visitors (N = 250) will be recruited from 2 public beaches in eastern Massachusetts. Beach site will be the unit of randomization. Follow-up assessment will occur at the end of the summer (1-month following intervention) and 1 year later. The primary outcome is average sunbathing time per week. The study was designed to provide 90% power for detecting a difference of .70 hours between conditions (standard deviation of 2.0) at 1-year with an intra-cluster correlation coefficient of 0.01 and assuming a 25% rate of loss to follow-up. Secondary outcomes include frequency of sunburns, use of sunless tanning products, and sun protection behavior.

**Discussion:**

Interventions might be improved by promoting behavioral substitutes for sun exposure, such as sunless tanners, that create a tanned look without exposure to UVR.

**Trial registration:**

NCT00403377

## Background

Ultraviolet radiation (UVR) from the sun is linked to more cancers worldwide than any other carcinogen.[1] Because skin cancer, the most prevalent cancer in the US [2], is largely attributable to UV exposure, it is largely preventable [3,4]. Prevention via avoidance of UVR seems like a simple solution, but rates of unprotected sun exposure remain quite high [5].

The belief that a suntan enhances physical attractiveness is a strong predictor of prolonged unprotected sun exposure [6-10]. Tanned images in the media play a big role in reinforcing the value of a tanned appearance [11]. The social norm for the attractiveness of a tanned appearance is difficult to change given the: 1) long history of this belief in the American culture, and 2) the number of tanned media images saturating the public [12]. Individuals who value being tan have been particularly resistant to educational messages about skin cancer prevention [13-15]. Comprehensive interventions that include education, sun damage imaging, free sunscreen, signage, and/or shade structures have been more successful at promoting sun protection than purely educational interventions although such interventions target outdoor recreationers [16-19], college students [20-22], or outdoor workers [23] and have not been examined among high risk groups such as intentional sunbathers. Beach-based interventions specifically targeting sunbathers with instant sun damage imaging which increases the saliency of sun-related skin damage have been somewhat effective [24,25]. Results have shown significant increases in sun protection, but mixed outcomes on sunbathing behavior. Beach visitors exposed to such interventions increase their use of sunscreen perhaps in attempt to tan 'safely' (i.e., without burning), but do not appear to reduce their sunbathing. This false sense of security might perpetuate further sun exposure.

A different approach to prevention would be to attempt to reduce sun exposure among sunbathers without battling their motivation to be tan. The promotion of behavioral substitutes, products that provide a safe, alternate route to achieving a tan, might preclude the need to sunbathe and result in reduced risk behavior. Increasingly popular over-the-counter sunless tanning products (e.g., self-tanners, spray tans) may be a substitute for sun exposure. Sunless tanning products come in the form of creams, foams, and sprays to be applied directly to the skin by the consumer or a professional. They contain dihydroxyacetone (DHA), a colorless vegetable-derived sugar that interacts with dead surface cells in the epidermis, thereby staining the skin a tan color [26]. The US Food and Drug Administration (FDA) approved DHA as a color additive for use in cosmetics in 1973 [27]. Sunless tanning products containing DHA can be obtained over the counter or professionally applied to the entire body in salons. The tan produced by these products cannot be washed off with soap and water like make-up or bronzers, which produce a darkening of the skin via water-soluble colorants [28]. Like a suntan, the effect of sunless tanning products is temporary, because as dead skin cells naturally slough off, the color fades and disappears within a few days unless the product is reapplied. Overall, sunless tanning products are associated with negligible health risks compared to direct sun exposure. DHA is both nontoxic [29] and hypoallergenic [30] but is not a significant source of sun protection. Although one might suspect that use of sunless tanning could lead to a false sense of security in terms of sun protection, cross-sectional studies have reported higher rates of sunscreen use among individuals who use sunless tanning [31-33]. Further investigation is needed to determine whether sunbathers would be willing to substitute sunless tanning products for a UV tan and if promoting sunless tanning reduces sunbathing.

### Research goals

In the present study, the efficacy of a multi-component beach-based intervention that features sunless tanning among female beach visitors will be tested. The intervention will promote sunless tanning as a substitute for sunbathing and include sun damage imaging and sun safety recommendations. The primary outcome will be sunbathing at 1-, and 12-months follow-up and a questionnaire-only control group will be employed for comparison. Secondary outcomes will include frequency of sunburns, use of sunless tanning products, and sun protection behavior.

## Methods

### Study design

This study is a cluster randomized trial design where data collection will occur simultaneously in two public beach locations located in eastern Massachusetts. The Departments of Public Works in the cities of Revere and Hull, Massachusetts have granted permission for study activities to be conducted at these beach sites. All study procedures and materials were approved by the University of Massachusetts Medical School's Institutional Review Board (IRB).

### Eligibility criteria

In order to capture the most natural, representative sample, few exclusion criteria will be imposed. Participants must be female and ages 18–75, English speaking, able to read at the 6^th ^grade level, and willing to participate. The proposed study will exclude individuals below the age of 18 because childhood sun protection and exposure behaviors are associated with different etiological influences than adult behavior, namely parental influences. Even though adolescents under 18 represent a high-risk group and may have some autonomy from parents regarding their sun protection behavior, the degree to which sunless tanning products appropriately address the variables contributing to their intentional sun exposure has not been addressed in the literature. Non-English speaking individuals will be excluded because the measures we propose to use have not been validated for use in other languages. Participants also have to be willing and able to provide at least two of the following: home phone, work phone, mailing address, home email, or work email, so they could be contacted for the follow-up assessments.

### Randomization

Beach locations (Revere and Nantasket Beaches in Massachusetts, US) will be randomly assigned to either intervention or control condition on each day of data collection during the month of July. Randomizing by beach location prevents contamination of conditions while holding weather conditions and day constant across groups. Weekend and weekdays will be balanced across locations and conditions, given that weekend beach visitors may have different characteristics than weekday beach visitors.

### Recruitment, screening process and informed consent

At both intervention and control beaches an unmarked tent will be set up in a central location during peak UV hours (11 a.m. to 4 p.m.) [34], on week and weekend days in which inclement weather was not forecasted. Research assistants will approach female sunbathers and ask them if they would like to participate in a study on sunbathing. If the sunbather is interested, eligibility will be assessed during this initial contact.

Eligible and interested individuals will then go to the study tent where participants will receive a verbal description of the study and be asked to read and sign an informed consent form. Upon providing informed consent, all participants, regardless of condition, will complete questionnaires. Participants, depending on the beach randomization, will then complete one of the two conditions.

### Outcomes and study measures

The primary outcome is sunbathing. Participants will be asked how much time they spent in the sun with the intention of getting a tan in the past month using a 9-point scale where 0 = never, 1 = once, 2 = twice, 3 = once a month, 4 = twice a month, 5 = once a week, 6 = twice a week, 7 = almost every day and 8 = everyday. The wording of this item is based on recommendations made by Glanz and colleagues 2008 [35]. However, the response options will be tailored to one month's time. This measure will be implemented at baseline, which will reflect sunbathing in the month previous to our contact with them at the beach, at 1-month follow-up to reflect sunbathing in the month following our contact with them at the beach, and at 12-months to reflect their sunbathing during one month of the following summer.

#### Sunburns

The number of times participants reported a red or painful burn that lasted a day or more in the past month will be assessed using a 6-point scale where 0 = not at all, 1 = once, 2 = twice, 3 = three times, 4 = four times and 5 = five times or more [35].

#### Sun Protection Behaviors

Consistent with Glanz and colleagues (2008) [35], participants will be asked to think about what they do when outside during the summer and then to respond to a series of questions about how often they use sunscreen, wear a shirt with sleeves, wear a hat, stay in the shade or under an umbrella, and wear sunglasses. For each, item, responses will be on a 5-point likert scale where 0 = never and 4 = always.

#### Sunless Tanning Behavior

Participants will be asked to read the following definition of sunless tanning: *Sunless tanning, also known as self-tanning or fake tanning, involves the application of creams, foams, sprays that dye skin a tanned color, or spray tans that you can get at a tanning business. These do NOT include bronzing powders and creams which can be washed off with soap and water*. They will then be asked the open-ended question, "how many times have you used sunless tanning products or spray-on tans in the last month?"

#### Indoor Tanning Behavior

Participants will be asked the open-ended question, "how many times have you used an indoor UV tanning device in the last month?"

### Intervention

#### Experimental condition

In addition to completing the questionnaires, participants randomized to the experimental condition will receive a brief intervention designed to increase sun protection behaviors, discourage sunbathing, and promote use of sunless tanning products as a substitute for sunbathing. First, participants will pose for three photographs: a standard Polaroid and two Polaroids using the UV filter that illuminates sun damage. Participants will have the opportunity to compare and take home the standard photo and one UV photo. During the development of the photographs, participants will be introduced to two products: sunscreen with SPF 30 and sunless tanning lotion. They will receive instruction on the proper use of each product and will be given a single use sample of each product to keep. The research assistant will guide the participant in applying the sunless tanner on a small portion of skin, so they can observe the skin-coloring effect firsthand. Participants will also be encouraged to use sunscreen on a daily basis, but especially while spending time in the sun. For the sunless tanning products, participants will receive instructions for proper use, be informed of the benefits of sunless tanning compared to sunbathing, be educated about the safety (and limitations, i.e., does not provide sun protection) of the product and its ingredients, and will view female models (research staff) with a sunless tan. Use of sunless tanning lotion will be encouraged as a healthy alternative to sunbathing rather than a supplement to sunbathing. The benefits to health and physical appearance of sunless tanning lotion as an alternative to sun bathing will be strongly emphasized as will the risks of continued sun exposure. Once participants view their photo, they will be instructed to post it in their home where it could serve as a reminder to use these products (e.g., medicine cabinet, near sundries, sports equipment, and refrigerator). Finally, participants in the experimental condition will receive an educational pamphlet that covers information about skin cancer detection, prevention, symptoms, and diagnosis, complete with graphic images of skin cancer lesions. A research assistant will review the main points of the pamphlet with the participant before giving it to the participant to take home.

#### Control condition

Participants randomized to the control condition will also complete study questionnaires. To balance the appearance of the intervention and time spent with interventionist, participants randomized to the control condition will be offered the opportunity to pose for and receive a free souvenir photograph to be taken with a standard Polaroid camera. Because intervention participants will receive free product samples for participation, control participants will also receive free product samples, but these will be of sundries that are irrelevant to skin cancer risk reduction (e.g., skin moisturizer, hair gel, chewing gum, breath fresheners). Study staff in the control condition will not receive sun safety education.

### Participant safety

Intervention staff will be extensively trained to provide accurate sun safety information. The participant may terminate participation at any time. Every effort will be made to ensure confidentiality. All data entered into computer files will be numerically coded with no names included. Participant files will be kept in a locked laboratory facility with access limited to personnel associated with the project. Participant files will be kept confidential and stored by the principal investigator.

Because this investigation poses relatively minimal risk to participants, a blinded Data and Safety Monitoring Board was not deemed essential. An internal Scientific Advisory Committee consisting of the principal investigator (PI), project director, project statistician, a consulting epidemiologist, an expert health behavior focus group leader, and a dermatologist, will be responsible for monitoring the progress, data, and safety of the trial. This committee will convene biannually to review progress, data, and safety.

Safety monitoring procedures will be documented in a standard protocol and overseen by the project director and consulting dermatologist. Study staff will contact the study dermatologist immediately to determine the best response to an adverse event involving sunless tanning or sunscreen products. The committee will review all events that have occurred and look for trends in the data. Adverse events, serious or otherwise, as mandated by the University of Massachusetts Medical School must be reported to the university IRB within 24 hours of the event. The PI will comply with this mandate and will also provide reports to the sponsor (NCI) and the FDA (when the adverse event is a result of sunscreens and sunless tanning products provided by the investigators). Summaries of adverse events are also required in annual reports by the University of Massachusetts Medical School. Any temporary or permanent suspension of this research (by the FDA or UMass IRB) will be reported by the PI to the NCI grant program director within 24 hours of that action.

### Retention

One month and 1 year following the initial beach contact, all participants will be contacted to complete follow-up questionnaires. All participants will be informed that they will receive a $10 gift card from their choice of stores for completion and return of the 1-month and $20 for the 1-year follow-up assessments. Additionally, participants will be informed that completion and return of surveys would enter them into a lottery to win a $500 gift certificate from the store of their choice.

At their initial contact on the beach, participants will be asked to rank order their preferred mode of contact for follow-up (i.e., email, mail, phone). For those preferring email, a link to online questionnaires will be sent via email. Hard copies will be sent via mail for those preferring mail, with instructions for completing and returning. The surveys will be completed via phone interview for those who prefer phone contact. The follow-up data collection team will begin with the first ranked mode. If no reply is received upon three attempts, the participant will be contacted via their second preferred mode of contact and so on. Three attempts will be made for each mode of contact or a total of 9 attempts will be made in all. Post cards will be sent to participants (who have not returned surveys) one and three weeks after they have been initially contacted. A database will be used to track all contacts and to tally response rates for each mode of contact. All contact information (phone, home address, and email) will be reviewed and updated at the one-month follow-up. During the 1-month follow-up, participants will be asked if they anticipate changes in contact information during the remaining 11 months of the study and to provide study staff with changes as necessary. These measures should increase rates of follow-up. All mailed surveys will include an addressed stamped envelope for participants use when returning surveys to researchers.

### Sample size considerations and statistical analyses

#### Sample size

The two-arm cluster randomized trial is designed to have at least 80% power at a 5% significance level to test the hypothesized intervention effects at 1- and 12-months on the primary outcome: average time spent sunbathing per week. Means and standard deviations from our previous investigation were used for sample size estimate. With the proposed analytic method, we estimated the required sample sizes for three testing scenarios, i.e., changes between baseline and 1 month, changes between baseline and 12 months, and the joint test involving all three time points (see Table 1). Sample sizes were calculated using the method developed by Frison [36]. Given the possibility that individuals recruited in the same day may show correlation between each other (intra-cluster correlation or ICC), we adjusted the sample sizes with assumed intra-cluster correlation coefficient of 0.01 with method of Garrett [37]. We anticipate a relatively low level of dependency should it exist (ρ = .01) given that individuals at the beach on one day may not be strongly different from individuals visiting the same location the next day. The estimated minimum sample sizes are presented in the Table 1 with the assumed parameters, specifically, the expected mean difference between the two arms after intervention is assumed to be 0.75 and 0.70 hours sunbathing per week at 1 and 12 months, the within-subject correlation is assumed to be 0.50 at 1 month and 0.45 at 12 months. These estimates allow for withdrawal and loss to follow up of 15% and 25% at 1 and 12 months.

**Table 1:** Sample size for three test scenarios for primary outcome: hours spent sunbathing per week

	Mean (SD)									
									
Test Scenarios	Control	Post-Intervention	Expected difference in mean	Within-subject correlation	No. of cluster (day)	Subject/cluster	Intra-cluster correlation	Minimum size/group	Dropout rate	Adjusted minimum total size
A (0–2 month)	2.10 (2.0)	1.35 (2.0)	0.75	0.50	16	12	0.01	94	0.15	**222**
B (0–12 month)	2.10 (2.0)	1.40 (2.0)	0.70	0.50	16	14	0.01	118	0.25	**314**
C (0–2–12 month)	2.10 (2.0)	1.38 (2.0)	0.72	0.50	16	9	0.01	66	0.25	**176**

### Primary and secondary hypotheses-analysis plan

#### Analytic plan

Intent to treat analyses (ITT) will be used to examine the primary outcome. All participants randomized will be included in analysis and analyzed by original group assignment [38]. Mixed effects regression modeling, implemented via SAS PROC MIXED that incorporated a random intercept trend, will be used since this analytic approach includes all participants that have data available on at least one time point [39]. Missing values will not be imputed or discarded, rather they will be treated as missing in the analysis. The analysis will include the linear time effect, the main effect of condition and the time and condition interaction to examine whether the experimental condition resulted in greater changes in sun behavior than the control condition over time. Age will be included as a covariate in the analysis. Chi-square analyses will be used to examine the differences between two conditions for the use of indoor tanning and sunless tanning at baseline and then at 1-month follow-up. Only participants with complete data at both time points will be included in the chi-square analyses.

#### Longitudinal analyses

Consistent with Consolidated Standards of Reporting Trials (CONSORT) guidelines, data on recruitment, participation rates, and drop-out will be collected and reported [40]. Should the frequency of missing data be less than 5%, baseline value carried forward will be conducted with proposed analyses. If the missingness becomes prevalent, the differences in demographic characteristics will be assessed between the completers and the noncompleters to identify possible non-differential missing patterns between intervention groups or demographic subgroups. If missing data appears to be not completely at random, a post-stratification method will be employed to address the issue. Otherwise, missingness will be assumed to be completely at random in the subsequent analyses. Since the data generated in this trial is longitudinal in nature, linear mixed models (LMM) or generalized linear mixed models (GLMM) will be applied to assess intervention effects on continuous and discrete outcomes, respectively [41]. A three-level LMM or GLMM (repeated observations, within subjects, within clusters) will be conducted with individual as the unit of analysis and cluster (day) entered as a random intercepts. Intervention indicators, as well as other key demographic confounders, will be included in the models as fixed effects. Subjects within each cluster will be considered independent from each other. For repeated observations within subjects, an unstructured correlation structure will be used. Finally, a separate set of analyses will be performed on participants who provided complete data. Should completer and ITT analyses yield similar results, the most conservative analysis will be reported. Should analyses yield different results, multiple analyses will be reported.

More specifically, the primary hypothesis will be tested using a linear mixed model with group (intervention, control) as a between subjects factor (fixed effect) and time (baseline, 1 month, and 1 year follow-up) as a within subjects factor. Sunbathing is the primary outcome variable. Secondary outcome variables including sun protection behavior, sunburns, and use of sunless tanning products will be examined in the same way. Because these dependent variables appear to be associated with different motivating variables, they are likely to be conceptually distinct and will be analyzed in separate models. Age will be entered as a covariate in both models because these factors are typically associated with the outcome variables [42]. Significant group by time interactions would be consistent with the primary hypothesis, such that the intervention group would engage in higher rates of sun protection and lower rates of sun exposure than the control group at 1- and 1-year follow-up, but not baseline. In exploratory analyses, ethnicity (White, nonwhite), and age will be entered as factors in the original model to determine differential response by these factors.

### Study operations, data management and tracking system

Dr. Pagoto will be responsible for oversight of study protocol and compliance. Ms. Oleski will be responsible for oversight of data tracking and study operations, and Dr. Ma will be responsible for oversight of data management and statistical analyses. At the time of data collection, research assistants will review participant responses on questionnaires with participants present. Skipped or incorrectly addressed items will be brought to the participant to correct. Under the direct leadership of the project director, project staff will be responsible for: 1) tracking participants to ensure that all necessary data are collected in a timely and efficient fashion, 2) developing and generating monitoring reports, 3) providing immediate timely and relevant feedback to project staff and leadership regarding the accuracy and precision of data, 4) writing all necessary programs and data screens for the collection of data, and 5) creating analytic data sets.

## Discussion

Almost two decades of study have clearly demonstrated that the belief that a tan improves appearance is one of the strongest predictors of sunbathing [6,11,43-45]. Of great concern is that most sunbathers are aware of their skin cancer risk but still continue to expose themselves to enough UV radiation to acquire a tan they believe will improve their appearance [46,47]. Interventions focused on promoting sun protection use without addressing tanning motivation are likely to be limited in their effect on this concerning group of tanners.

The media together with peer influences are converging forces that promote the value of a tanned appearance [11]. The increasing popularity of spray tanning may perpetuate the social norms about tanned skin, potentially undermining efforts to promote pale skin as a beauty standard. Reversing the social norm toward pale skin might not be the most effective avenue to reducing skin cancer risk among sunbathers. Attempting to convince sunbathers to give up a strong, immediate reinforcer (suntan) with threats of indefinite long term health consequences (skin cancer), and no alternatives is a difficult task. An alternative approach is to make sunbathers aware of healthy and safe alternatives to sunbathing, allowing them to make a healthy decision that is consistent with their goals and concerns.

Behavioral economics theory represents a framework for understanding health behavior decisions based on alternatives. This theory has been successfully applied to dietary intake [48], smoking [49] and substance abuse [50]. It posits that: 1) as the cost (time, money, physical or emotional risks, etc) of engaging in a behavior rises, the rate of behavior declines (e.g., as cigarettes become more expensive, rates of smoking have declined), and 2) as behavioral substitutes become available, the rate of the target behavior also declines, especially if the relative cost of the substitute is less than that of the target behavior (e.g., with the introduction of sugar substitutes into the marketplace, the rising consumption of diet soda is associated with reduced consumption of regular sodas) [51]. Behavioral economics includes a number of principles that may be helpful for designing effective skin cancer risk change messages. First, delayed outcomes (e.g., long-term health) have less impact on behavior than immediate outcomes (e.g., having tanned skin). Changing behavior requires that the immediate outcomes for not engaging in the behavior or for engaging in alternative behaviors outweigh the immediate outcome of engaging in the behavior. Second, behaviors with highly desired outcomes (e.g., tanned appearance) but limited alternatives are more resistant to change even as the cost rises. For example, despite the current dramatic increases in gas prices, demand remains fairly unchanged. Similarly, if no alternatives exist, rising skin cancer rates might not be accompanied by reduced rates of sunbathing. Finally, non-mutually exclusive behaviors such as UV and sunless tanning can be affected by changing their relative perceived costs. Therefore, as the costs of sunbathing (e.g., perceived risk for skin cancer, skin damage, burns, etc) accumulate, the use of alternatives such as sunless tanning, that produce the same outcome with fewer costs, should increase.

The present study tests the efficacy of a sun safety intervention based on the principles of behavioral economics theory. Sunless tanning products will be promoted among beach visitors as a safe alternative to sunbathing. Participants will also be made aware of the sun damage on their skin via sun damage imaging and be given information on skin cancer risks, consequences, and methods of protection. The promotion of sunless tanning within the context of a skin cancer prevention intervention has potential to impact sun exposure by giving sunbathers a healthy alternative to achieve a tan.

## Competing interests

The authors declare that they have no competing interests.

## Authors' contributions

SP conceived, designed and obtained funding for the study. YM and JO participated in the study's design. JO was involved in the study's coordination. YM and KS participate in the study's data management. JO is involved in participant safety and consultation. SP, KS, YM, JB and PM drafted the manuscript. All authors read and approved the final manuscript.

## Pre-publication history

The pre-publication history for this paper can be accessed here:


